# Musicians’ pursuit of expertise-related goals is characterised by strategic regulation of functional and counterproductive affect

**DOI:** 10.3389/fpsyg.2024.1407303

**Published:** 2024-09-04

**Authors:** Gerard Breaden Madden, Steffen A. Herff, Scott Beveridge, Hans-Christian Jabusch

**Affiliations:** ^1^Institute of Musicians’ Medicine, University of Music Carl Maria von Weber, Dresden, Germany; ^2^Sydney Conservatorium of Music, University of Sydney, Sydney, NSW, Australia; ^3^The MARCS Institute for Brain, Behaviour and Development, Western Sydney University, Sydney, NSW, Australia

**Keywords:** musical practice, musicians, mastery, emotion regulation, anger

## Abstract

**Background:**

Emotion regulation is an important part of effective goal pursuit. Functional accounts of emotion regulation suggest that the attainment of challenging goals may be supported by regulating emotions which promote utilitarian over hedonic outcomes. When pursuing the challenging, long-term goal of acquiring expert musical skills and knowledge, musicians may wish to prioritise whichever emotions are most conducive to attaining this goal, even if those emotions are not necessarily positive.

**Methods:**

Via an online questionnaire, musicians (*N* = 421) answered questions concerning their musical experience and their expertise-related practice goals. They also reported how strongly they experienced different emotions during practice, and how strongly they desired to either increase or decrease the intensity of those same emotions. Data were analysed using inferential frequentist statistics and Bayesian mixed effects models. Evidence ratios (ER) > 19 were considered strong evidence in favour of an effect.

**Results:**

Our analysis showed that musicians experienced and desired strong levels of positive emotions in their practice. In addition, they reported greater desire to intensify positive compared to negative emotions [*paired t* (420) = 58.13, *p* < 0.001]. Our Bayesian mixed effects model provided strong evidence that greater desire to intensify anger increased the probability that an observation derived from a musician with stronger expertise-related goals [Est = 0.70; Odds (Est > 0) > 9,999]. In addition to anger, higher levels of expertise-related goals were increasingly predicted by less strong desire to intensify guilt and gloom and greater desire to reduce downheartedness (all ER > 19).

**Discussion:**

Overall, musicians had a strong, general desire to intensify positive emotions during their musical practice. However, musicians with higher levels of expertise-related goals increasingly indicated a nuanced approach regarding how they desired to regulate certain negative emotions. Findings suggest that musicians engage in selective and sophisticated emotion regulation behaviour that aligns with their long-term commitment to develop musical expertise. They may prioritise emotions which may be functionally beneficial, whilst avoiding emotions which may be counterproductive or undermine efforts. Findings from this study contribute to our understanding of expertise-related, domain-specific emotion regulation behaviour and may inform the design of prioritised musical practice strategies.

## 1 Introduction

An important goal for many musicians is the acquisition of expert musical skills and knowledge—sometimes referred to as *mastery*. Mastery is a long-term and technically demanding ambition that requires many years of sustained, deliberate, and varied practice (Ericsson et al., 1993; Lehmann and Ericsson, 1998). Progress during musical practice is neither linear nor assured, and during their training musicians may encounter challenges which can stymie motivation and satisfaction. To persevere despite such challenges and to optimise performance and attain their goals, musicians may engage in goal-related emotion regulation during musical practice (Breaden Madden and Jabusch, 2021).

Emotion regulation is a constellation of affective, behavioural, and cognitive processes through which we influence how and when we experience and express emotions (Gross, 2015). These processes help us to function effectively and cope with situational demands by acting on our motivation, proactivity, and learning styles (Foo et al., 2009; Kobylinska and Kusev, 2019; Mazur and Laguna, 2019). As important outcomes in music such as success and advancement usually depend on demonstrations of expert skills (in performances or competitions, for example), regulating emotions which promote mastery can be seen as an important and necessary component of any serious plan to develop expert musical skills and knowledge.

Research increasingly adopts a functional perspective of emotion regulation, recognising that emotions are neither wholly good nor bad *per se*, rather that they can be more-helpful or less-helpful depending on the demands of a given situation or goal (Lane, 2012; Lench et al., 2023). According to this perspective, emotions possess different degrees of *hedonic* and *instrumental* potential, depending on how and when they are experienced. The hedonic potential relates to an emotion’s capacity to elicit immediate and pleasurable experiences, whilst the instrumental potential relates to how an emotion can support utilitarian, outcome-related benefits (Tamir et al., 2008; Lane et al., 2011). Take, for example, positive emotions; these may possess both hedonic *and* instrumental potential—not only are they pleasurable to experience, but they also facilitate outcome-related benefits, such as better decision-making and creativity (Carnevale and Isen, 1986; Estrada et al., 1994) as well as better motor performance in activities such as musical scale playing (Spector et al., 2014). Perhaps as a reflection of these benefits, it is generally assumed that individuals are motivated to experience positive emotions in most contexts and to support most activities (e.g., Diener, 2000; Larsen, 2000; Tsai et al., 2007). In certain circumstances however, a positive outcome may be much more valuable to an individual than a positive emotional experience (Tamir et al., 2008). In fact, if a particular outcome is *especially* important or desirable, then an individual may prioritise whichever emotions are most conducive to that outcome and avoid whichever emotions are seen as counterproductive (Lane, 2012). In such cases, the instrumental potential of an emotion is more valuable than the hedonic potential. Furthermore, if an important outcome is increasingly challenging or difficult to attain, an individual may even wish to maximise the instrumental potential of their emotional state and minimise the hedonic by prioritising emotions which are not necessarily positive (Lench et al., 2023).

To wit, although positive emotions can be functionally beneficial *as well as* pleasant, negative emotions can be functionally beneficial but are *not* typically pleasant to experience. This suggests that negative emotions may be particularly useful when the desire to attain a demanding goal is high (Breaden Madden and Jabusch, 2021). There is increasing empirical evidence to support this perspective. For example, Lane et al. (2011) showed that most runners preferred to feel good in the lead-up to a race. However, a minority reported that they preferred to intensify negative emotions such as nervousness instead of positive emotions. This preference to intensify negative emotions was liked to meta-emotion beliefs that these emotions would benefit their competitive performance. In a video game scenario where participants completed competitive tasks, anger was shown to facilitate better performance compared to neutral or positive emotional states (Tamir et al., 2008). Similarly, when compared to other states (neutral, sadness, amusement, and desire) anger was showed to produce better outcomes when participants completed different technical and demanding tasks such as solving puzzles, winning prizes, and avoiding challenges (Lench et al., 2023).

Musical practice may not always be an enjoyable experience (Ericsson et al., 1993). Many hours spent refining demanding playing techniques, honing precise motor sequences, or memorising complex compositions may well be at times challenging and even unpleasant. Many musicians persevere in their practice nonetheless, suggesting that there are benefits to practice beyond the immediate and hedonic. Musicians who are particularly serious in their pursuit of musical expertise may then be willing to experience emotions which do not necessarily emphasise immediate hedonic reward in their practice (Breaden Madden et al., 2023). As emotions can arise in particular during moments of challenge, the issue of proper emotion regulation is a matter of importance (Gross, 1999) as it may determine how successful our efforts are. The aim of this research is to investigate the relationship between musicians’ expertise-related practice goals and their desire to regulate different emotions during musical practice.

## 2 Methods

### 2.1 Materials and procedure

Ethical approval for this study was provided by the responsible institutional review board (see the section “Ethics Statement”). Participants provided informed consent to take part before completing an online questionnaire in English. Those aspects of the questionnaire relevant to the current analysis are described below. An abridged version of the questionnaire is included in Supplementary Appendix A (i.e., excluding modules which are based on already published research). See Breaden Madden and Jabusch (2021) for a complete description of all questionnaire modules including those not reproduced here.

#### 2.1.1 Demographics and musical expertise

Participants reported standard demographics and information related to musical expertise including: the age at which they began making music (Age of Commencement; AoC), the total number of years making music (Years of musical Practice: YoP), Cumulative Life Practice time (CLP; derived from retrospective self-reported year-by-year practice hours), status as a student or professional musician, and primary musical instrument family.

#### 2.1.2 Emotions in musical practice *(seven-point Likert scale; 1 = not at all, 7 = a great deal)*

Participants completed three emotion scales based on the UWIST Mood Adjective Checklist (Matthews et al., 1990). The first scale concerned how strongly musicians typically experienced different emotions during their musical practice (referred to hereafter as actual emotions). In the second and third scales, musicians indicated how strongly they desired to either intensify or reduce these same emotions during their musical practice, respectively. The three scales utilised terms derived from the emotion circumplex model (Russell, 1980) and assessed high- and low-arousal positive emotions (*Happiness, Calmness*), high-arousal negative emotions (*Anger, Anxiety*), low-arousal negative emotions (*Gloom, Guilt, Downheartedness*), and emotions that vary in terms of energetic-arousal (*Energy, Nervousness,* and *Sluggishness*).

#### 2.1.3 Mastery Goal Orientation *(seven-point Likert scale; 1 = very untrue of me, 7 = very true of me)*

Mastery Goal Orientation was established via Principal Component Analysis (PCA) based on musicians’ responses to questionnaire items about musical practice activities and attitudes. This factor included the items “*It is very important to me to continue to perfect my musical and technical abilities*.”, “*I practice difficult techniques or pieces until I have mastered them”*, and “*I practice so that I can play a piece exactly as I think it should be*.” Musicians with higher scores on this factor possessed stronger Mastery Goal Orientation, and vice versa. See Breaden Madden and Jabusch (2021) for a complete description of this factor and the associated PCA procedure.

### 2.2 Participants

Four hundred and twenty-one musicians (Female/Male = 254/167) participated in this study. They were recruited from music institutions worldwide including orchestras, conservatoires, and music universities. The majority were recruited from: United States of America (113), United Kingdom (72), Germany (64), all other participating countries (< 50 each).

These musicians commenced making music at a median age of 7 years (minimum/maximum = 2/19), had a median age of 23 years (18/68) at the time of the study, a median YoP of 16 years (6/54), and a median CLP of 8,662 h (391/80,853). The majority of musicians in this sample indicated that they were student (301) rather than professional musicians (120). Bowed string instrumentalists were the most numerous in this sample (105), followed by keyboard players (81), woodwind (67), voice (62), brass (51), plucked string (37), and percussion (18).

### 2.3 Statistical analysis

First, we examined musicians’ actual and desired emotions in musical practice using descriptive frequentist statistics and *t*-tests. Second, we deployed a Bayesian mixed effects model utilising musicians’ desire to intensify or reduce the level of these practice-related emotions as predictors of Mastery Goal Orientation. The model controlled for Sex, Age, AoC, CLP, YoP, status as a student or professional musician, and primary musical instrument family. In addition, the model was fitted with a random effect for participant and a weakly informative prior (t-distribution with a mean of 0, standard deviation of 1, and 3 degrees of freedom; see Gelman et al., 2008). The chosen prior and evidence ratio reference are established in research on music psychology and music perception (e.g., Dobrowohl et al., 2019; Herff et al., 2020; Cecchetti et al., 2021; Breaden Madden et al., 2023, among others).

To investigate the main effect of each emotion, we report hypothesis tests which evaluate the evidence of a given effect to be smaller or larger than zero (Evidence Ratio; ER) as well as the coefficient estimate (Est.) and the error within each estimate (Est. Error). ERs > 19 are considered strong evidence for an effect (see Milne and Herff, 2020) and are denoted with an *.

## 3 Results

### 3.1 Emotions in musical practice

**Figure 1:**
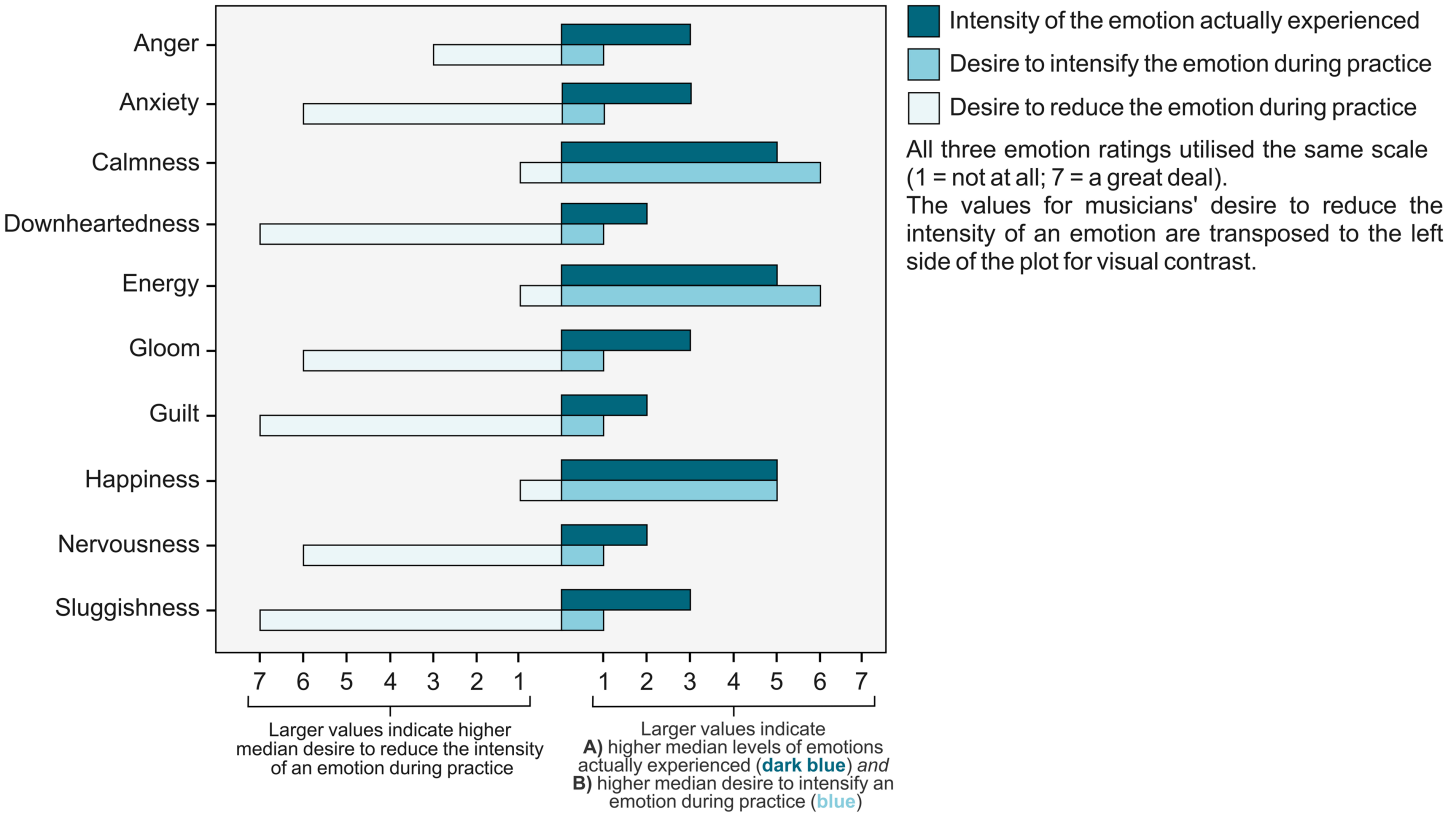
Median levels of actual and desired emotions in musical practice. Figure 1 is based on median data originally published in Breaden Madden et al. (2023).

Positive emotions such as happiness and calmness were typically experienced at moderate/high levels during musical practice. Musicians reported strong desire to intensify positive emotions, coupled with very little/no desire to reduce them. Negative emotions such as downheartedness and anxiety were typically experienced at low/moderate levels during practice. Musicians reported very little/no desire to intensify negative emotions in addition to generally strong desire to reduce them. See Figure 1 for an overview of these data.

Overall, musicians showed stronger desire to intensify positive emotions than negative ones [*paired t* (420) = 58.13, *p* < 0.001; 95% CI (−13.36, −12.07)]. They also indicated stronger desire to reduce negative emotions compared to positive ones [*paired t* (420) = −36.87, *p* < 0.001; 95% CI (28.87, 31.51)]. *T*-test findings were originally reported in Breaden Madden et al. (2023).

### 3.2 Predicting Mastery Goal Orientation

Our Bayesian model featured Mastery Goal Orientation as a target/outcome variable and included musicians’ desire to regulate (intensify or reduce) emotions as predictors. Using this model, we identified strong evidence for several effects (where strong evidence refers to effects with an ER > 19: denoted with *). Specifically, greater desire to intensify anger increased the probability that an observation derived from a musician with stronger Mastery Goal Orientation (Est. = 0.70, Est. Error = 0.19, ER > 9999*). In other words, musicians desired an increase of 0.70 standard deviations in anger for each 1 standard deviation of Mastery Goal Orientation above the mean.

**Figure 2:**
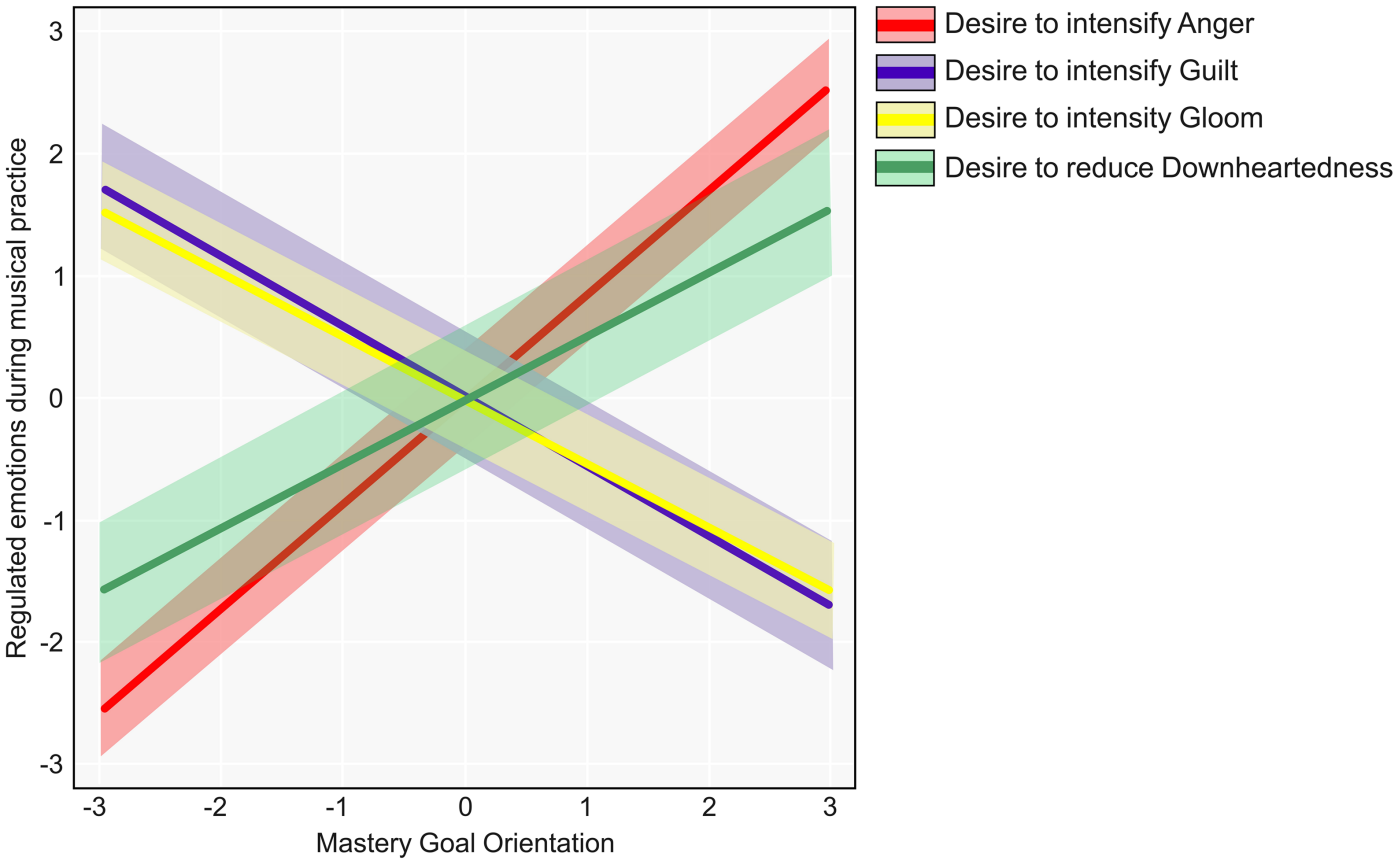
Marginal effects of the desire to regulate practice-related emotions on predicted level of Mastery Goal Orientation. Line colour indicates each emotion. Bands represent 95% Credible Intervals (CI). Values are standardised (Mean = 0, SD = 1).

In addition, stronger Mastery Goal Orientation was predicted by less desire (indicated by a negative estimate coefficient) to intensify guilt (Est. = −0.50, Est. Error = 0.25, ER = 40.92*) and gloom (Est. = −0.44, Est. Error = 0.20, ER = 70.02*), as well as greater desire to reduce the intensity of downheartedness (Est. = 0.52, Est. Error = 0.31, ER = 19.85*). Figure 2 depicts the marginal effects of these emotions on model predictions for Mastery Goal Orientation. An upward line trajectory corresponds to a positive coefficient estimate, and vice versa. Only those emotions for which we identified strong evidence (ER > 19) are shown on this plot. Although musicians’ desire to either intensify or reduce emotions were measured on separate scales, all effects for which ER > 19 are combined in one plot.

## 4 Discussion

Our findings show that musical practice typically involved strong positive actual emotions, and that musicians desired to intensify positive over negative emotions in support of their practice goals (see Section 3.1). This suggests not only that musical practice is a positive experience in general—but also that musicians want it to be. In addition, our findings show that when musicians indicated increasingly greater desire to intensify anger, reduce downheartedness, and less desire to intensify gloom and guilt, they reported strong Mastery Goal Orientation.

Approximately 70% of our emotional experiences are thought to be traceable to goal-related events (Oatley and Duncan, 1994). Although individuals utilise positive emotions to serve goal-specific functions more often than unpleasant emotions, the purposeful (rather than reactive) use of the latter leads to more beneficial outcomes (Weidman and Kross, 2021). This premise is applicable to our current findings. With higher levels of Mastery Goal Orientation, musicians increasingly indicated nuance regarding how they desired to regulate different specific negative emotions. In other words, they did not indicate a desire to intensify negative emotions *as a whole*, nor reduce them *as a whole.* This nuanced desire for specific negative emotions was observed in conjunction with musicians’ general desire to intensify positive emotions during musical practice (at a descriptive level: see Figure 1). The selective desire to regulate specific negative emotions can perhaps be seen as a fastidious strategy which characterises Mastery Goal Orientation. We view it as a potential consequence of musicians’ exposure to regular practice-related challenges when practicing specifically with the aim of developing expertise. These findings build on those of Breaden Madden and Jabusch (2021). They showed that some musicians who possessed stronger expertise-related goals sought a mixed emotional state (combining both positive and negative emotions) to support their practice activities. These same musicians held the belief that unpleasant emotions such as anger may be beneficial to their practice.

### 4.1 Functional and counterproductive qualities of the emotions regulated during mastery-focused musical practice

Anger is a prototypical negative emotion, often conflated with aggression/violence and contexts in which rage impairs one’s judgement. Despite its low hedonic appeal, anger is understood to possess motivational and assertive properties, making it a potentially valuable tool for musicians who set challenging practice-related goals. At a neurophysiological level, anger is associated with increased left- and decreased right-prefrontal cortex activation. This asymmetric activity has been suggested to be connected to motivational direction more so than emotional valence (Harmon-Jones and Sigelman, 2001; Carver and Harmon-Jones, 2009). Indeed, anger is increasingly framed as an appetitive and approach-related emotion. It functions as an inwardly directed signal to overcome obstacles, leading to greater goal-related planning and faster execution of predetermined behaviour (Maglio et al., 2014; Khan et al., 2019).

As outlined earlier, musical practice can be a regularly challenging activity. Given that anger does not relate to goal attainment when challenges are low (Lench et al., 2023), invoking anger may equip musicians with the assertive energy necessary to practice effectively under challenging conditions. On the other hand, however, practising can advance one’s abilities and knowledge, and this potential for advancement may be a positive experience in and of itself. Taken together, these points suggest that musical practice may involve anger in combination with positive emotions. Interestingly, Harmon-Jones et al. (2009) suggested that positive emotions and anger may overlap in situations which involve approach motivation (such as musical practice). Furthermore, they suggested that contrary to expectation, positive emotions may even be increased in situations that feature anger. This may account for why *some* musicians from our study had stronger desire for anger, whilst musicians *overall* indicated a general desire for positive emotions. Musical practice is presumably then much more than a collection of musical and technical activities and developing skills and knowledge. It can also be seen as an emotionally rich activity encompassing a spectrum of both positive and negative emotional experiences. In so far that musical practice is a regular source of potential challenge, those who desire musical expertise may believe that a combination of anger and general positive emotions may be a functionally beneficial choice to help them attain challenging goals.

Unlike anger and positive emotions, low-arousal negative emotions such as guilt, downheartedness, and gloom may be counterproductive for musicians who concentrate on developing expert musical skills and knowledge. If an individual experiences too much of such emotions, it can hinder the successful regulation of other, more productive emotions (Kalenska-Rodzaj, 2018) and can disrupt effective goal-related planning and the processing of reward (Höflich et al., 2019). Guilt, for example, can lead to self-punishment, avoidance of challenging situations, and unhealthy patterns of behaviour if an individual overcompensates for perceived wrongdoing. In neurophysiological terms, guilt has been associated with an activation in several brain regions including the ventromedial prefrontal cortex and the subgenual cingulate cortex (Zahn et al., 2009). These areas are involved in processes such as value-based decision making and the processing of self-knowledge (Hiser and Koenigs, 2017). Increased or prolonged experiences of guilt may lead to neuroplastic changes in these brain areas, such as those observed in individuals suffering from depression (for a review, see Rădulescu et al., 2021). Such changes may impact how an individual functions in challenging situations and may contribute to a greater focus on risk-avoidance rather than desired outcomes. It may be the case that deliberate down-regulation of guilt is conducive to more effective musical practice as a musician is better equipped to set appropriate goals and adapt their personal strategies for improvement when guilt is at a low level.

Not unlike guilt, gloom and downheartedness are characterised by counterproductive feelings of hopelessness, passivity, and negative assessments of one’s abilities. Excessive levels of these emotions may contribute to diminished motivation and neglect of one’s musical activities. We see it as encouraging that musicians reported generally low actual levels of these emotions in their practice (as with most other negative emotions assessed here). The reported desirability of these emotions was also low. Musicians desired to either maintain these emotions (less desire to intensify guilt and gloom) or suppress them (greater desire to reduce downheartedness). Importantly, they indicated no desire to intensify them.

Experiencing the low-arousal and typically unpleasant qualities of guilt, gloom, and downheartedness may undermine the disciplined and focused mindset required to achieve musical mastery. Anger, on the other hand, may be functionally beneficial by motivating persistence despite challenges. Musical training is thought to foster better affective sensitivity (Gujing et al., 2019) and the ability to process and convey emotional information (Lehmann and Ericsson, 1998). Furthermore, musicians demonstrate better active coping and planning skills compared to non-musicians (Soudi and Aminabhavi, 2015). The connection between Mastery Goal Orientation and the nuanced desire for negative emotions speaks to the possibility that pursuing musical expertise through practice may promote sophisticated meta-emotion knowledge. Such knowledge may help musicians to prioritise and deploy specific emotions based on their hedonic/instrumental qualities and potential goal-related benefits. Musicians may utilise such knowledge to better pursue specific outcomes in their practice and optimise their performance.

### 4.2 Directions for development

This study adds to the relatively limited body of literature on emotion regulation in contexts centred on specific activities rather than to support specific social purposes (Lench et al., 2023). Our findings contribute to the understanding of how the functional/utilitarian potential of emotions may account for how musicians wish to feel during their practice. Data collection took place during the COVID-19 pandemic. This situation may have had an impact on participants’ mood, especially given that many music institutions suspended operations during this time. We acknowledge that this situation may have had an impact on our findings.

Future research could focus on whether desired emotions vary when musical goals other than expertise are pursued, or when individuals pursue expertise in non-musical domains. From a neurophysiological perspective, investigating desired emotions may clarify the potential that goal-related emotion regulation during musical practice has to induce neuroplastic change. As learning strategies evolve and diversify as a musician gains experience over time (Lehmann and Papoušek, 2003), researchers could investigate variation in the desirability of different emotions over the course of musical training, as well as strategies to evoke specific desired emotions used by musicians who differ in experience or by those who desire expertise in multiple instruments. Finally, as this study did not evaluate practice outcomes, it may be beneficial to investigate whether and how objective short-term and long-term practice results and development of expert musical and instrumental skills depend on the regulation of specific desired emotions including positive emotions and anger.

## 5 Conclusion

Both positive emotions and anger may have the capacity to benefit musicians who aim to develop greater musical expertise. Musicians who reported higher levels of expertise-related goals indicated greater nuance regarding their desire for negative emotions in their practice. This included an avoidance of low-arousal negative emotions which may be counterproductive to their efforts. Such nuance suggests sophisticated emotion regulation knowledge which strikes a balance between the hedonic and instrumental potential of emotions. These findings contribute to our understanding of domain-specific, expertise-related mindsets. This and future research on this topic may also aid the design of individualised practice strategies concerning how functional and counterproductive emotions are regulated.

## Data availability statement

The public availability of this data set is not covered by the ethics application and approval provided for this study (State Chamber of Physicians of Saxony [IRB: 425931; Application Reference: EK-BR-73/19-1]). Requests to access the data set of this study should be directed to GBM.

## Ethics statement

The studies involving humans were approved by the Institutional Review Board of the State Chamber of Physicians of Saxony, Germany (IRB: 425931; Application Reference: EK-BR-73/19 -1). The studies were conducted in accordance with the local legislation and institutional requirements. The participants provided their written informed consent to participate in this study.

## Author contributions

GBM: Conceptualization, Data curation, Formal analysis, Investigation, Methodology, Project administration, Resources, Software, Supervision, Visualisation, Writing – original draft, Writing – review & editing. SAH: Formal analysis, Methodology, Investigation, Writing - review & editing. SB: Writing – review & editing. H-CJ: Investigation, Writing – original draft, Writing – review and editing.
