# Supplementary material for: Musicians’ pursuit of expertise-related goals is characterised by strategic regulation of functional and counterproductive affect
Source: Front Psychol. 2024 Sep 4;15:1407303. doi: 10.3389/fpsyg.2024.1407303 (PMC11408472; doi:10.3389/fpsyg.2024.1407303)
Supplement: Supplementary file 1 [file Data_Sheet_1.pdf]

Supplementary material for the article:

Musicians' pursuit of expertise-related goals is characterised by strategic regulation of functional and counterproductive affect.

Gerard Breaden Madden[1*], Steffen A. Herff[2,3], Scott Beveridge[1], Hans-Christian Jabusch[1]

[1]Institute of Musicians' Medicine, University of Music Carl Maria von Weber, Dresden, Germany
[2]Sydney Conservatorium of Music, The University of Sydney, Sydney, NSW, Australia
[3]The MARCS Institute for Brain, Behaviour, and Development, Western Sydney University, Sydney, NSW, Australia

***Corresponding author:** gerard.madden@hfmdd.de

Appendix A: Abridged* Emotion Regulation Questionnaire

*Certain modules from this questionnaire are not reproduced here as they are taken/adapted from existing published measures. Please see Breaden Madden and Jabusch (2021) for a complete description of the questionnaire including those modules.

Section 1: Demographics and Musical Experience

Age: [ ] **years**

Gender: Male ☐ Female ☐ Other ☐

Please tick the box that most accurately describes the type of musician you are:

Professional ☐ Amateur ☐ Music Student ☐ Other (please describe): [ ]

At what age did you start playing music? [ ] **years**

At what age did you begin taking music lessons? [ ] *I have never taken music lessons* ☐

How often do you practice or rehearse music in the company of a teacher? [ ]
(1 = never; 7 = all the time)

How often do you practice or rehearse music in the company of other musicians? [ ]
(1 = never; 7 = all the time)

Please think about the time when your music practice is / was at its highest.
What is / was your instrument at this time?

Woodwind	*(e.g., flute, clarinet, oboe)*	☐
Brass	*(e.g., trumpet, trombone, tuba)*	☐
Percussion	*(e.g., drums, timpani, xylophone)*	☐
Keyboard	*(e.g., piano, organ, harpsichord)*	☐
Bowed string	*(e.g., violin, cello, viola)*	☐
Plucked string	*(e.g., guitar, bass, harp)*	☐
Voice		☐
Other *(please write the name of your instrument):*		[ ]

Since starting to play this instrument, have there been any breaks where you did not play your instrument? Yes☐ No☐
if No, please leave the following question blank

If there have been breaks, how long have these breaks been?
(*Please indicate your age at the beginning of each break and the duration of the break, in months)*
Age: [ ] Duration of break (in months): [ ]
Age: [ ] Duration of break (in months): [ ]
Age: [ ] Duration of break (in months): [ ]
Age: [ ] Duration of break (in months): [ ]

How many days each week are you currently working on your main instrument (either practicing and/or playing)? 1 ☐ 2 ☐ 3 ☐ 4 ☐ 5 ☐ 6 ☐ 7 ☐

On average, how many hours per day do you practice and/or play your instrument? [ ] **hours**
(For practice times less than one hour, please indicate the average time as 0.25 hours, 0.5 hours, etc.)

Please enter your average <u>weekly</u> practice and/or playing time for each age range *(in Hours)* [ ] **hours**
(Remember to calculate this for your entire week – For example, if you practice 2 hours every day, then your weekly practice is: 2 hours x 7 days = 14 hours)

**Age**	Under 6	6-9	10-13	14-16	17-19	20-22	23-25	26-28	Over 28
**Hours per week**	[ ]	[ ]	[ ]	[ ]	[ ]	[ ]	[ ]	[ ]	[ ]

Do you play (an)other instrument(s) in addition to your main instrument? If yes, please tick the group that includes this instrument *(Multiple answers are possible, please tick all that apply)*

Woodwind	*(e.g., flute, clarinet, oboe)*	☐
Brass	*(e.g., trumpet, trombone, tuba)*	☐
Percussion	*(e.g., drums, timpani, xylophone)*	☐
Keyboard	*(e.g., piano, organ, harpsichord)*	☐
Bowed string	*(e.g., violin, cello, viola)*	☐
Plucked string	*(e.g., guitar, bass, harp)*	☐
Voice		☐
Other *(please write the name of your instrument):*		[ ]

How many years have you played this other instrument(s) in total? [ ] **years**

Please rate how active you are in each style of music (either as part of your practice, playing or rehearsal) *(1 = Extremely inactive, 7 = Extremely active)*

Blues	☐	Jazz	☐
Classical	☐	Opera	☐
Contemporary Classical (post 1950's)	☐	Pop	☐
Country	☐	Punk	☐
Dance / Electronica	☐	Rap / Hip-Hop	☐
Folk	☐	Rock	☐
Funk	☐	Soul / R&B	☐
Heavy Metal	☐		

How much do you agree with the following statements about your practice?
(1 = Strongly disagree, 7 = Strongly agree)

The material I practice is decided by someone other than me (e.g., teacher, parent, conductor, manager)	☐
When I practice, I usually feel in control of the material I am practicing	☐
I really enjoy the experience of music practice	☐
I am totally focused on what I am doing in practice	☐

Section 2: Goals in Music Practice

Please think about when you practice or rehearse music alone, without a teacher or other musicians. Using the scale below, please rate each statement according to how much it applies to you. *(If you are a vocalist, please include voice as an instrument)*
(1 = Very untrue of me, 7 = Very true of me)

I play my instrument for relaxation/recreation	☐
I have to see success/progress, otherwise I don't enjoy playing my instrument	☐
It is important for me to be challenged by my teacher	☐
I enjoy practicing so that I can play a piece exactly as I think it should be	☐
I often compare myself with others that play my instrument	☐
After a long day, I don't put as much effort into my playing	☐
I enjoy practicing a difficult technique or piece until I have mastered it	☐
It is important for me to continue to perfect my musical and technical abilities	☐
I enjoy playing my instrument because it helps me to relax and forget everything around me	☐
I don't always have to play very difficult pieces. The main thing is that they sound nice	☐
Learning new things and acquiring new skills on my instrument is a challenge for me	☐
It is important to me that I get along well with my teacher	☐
At my age, you don't have to take practice too seriously. After all, I only play for myself and I don't want to become a professional musician	☐
I prefer to play pieces that I am good at. I find it tedious, strenuous and unsatisfying attempting new pieces or technical exercises	☐

The following statements concern your beliefs about how emotions can impact your music practice. Using the scales below, please write a number in each box below that best represents your opinion about each statement. *(1 = Strongly disagree, 7 = Strongly agree)*

I usually feel I have to change my emotions in order to get the most out of my practice ☐
I know what emotions will help me get the most out of my practice ☐
I pay attention to my emotional state during my practice ☐
The emotions I usually feel in practice don’t help me get the most out of my practice ☐
I must have the right emotional state in order to get the most out of my practice ☐
I actively seek to experience emotions that will help improve my practice ☐
I am usually able to change my emotions to be in the right mindset for music practice ☐
When I finish practicing, I usually feel that my practice has gone well ☐
Music practice is best when I am feeling positive ☐
I don't always have to feel good to practice effectively ☐
I cannot practice well unless I am feeling positive ☐
Negative emotions such as anger can help improve my music practice ☐
I believe that the emotional state I have during practice is useful for me to achieve my goals ☐
I work equally as hard in practice, regardless of how practice makes me feel ☐

Section 3: Emotion in Music Practice

Think about when you practice music alone. Using the scale below, please rate how strongly you actually experience each of the following emotions during your typical music practice.
(1 = Not at all; 7 = Very strongly)

Happiness ☐
Energy ☐
Calmness ☐
Gloom ☐
Guilt ☐
Sluggishness ☐
Anxiety ☐
Downheartedness ☐
Nervousness ☐
Anger ☐

Based on your previous experience, how much you would like to INCREASE each of the following emotions to get the most out of your music practice?
(1 = Not at all; 7 = A great deal)

Happiness ☐
Energy ☐
Calmness ☐
Gloom ☐
Guilt ☐
Sluggishness ☐
Anxiety ☐
Downheartedness ☐
Nervousness ☐
Anger ☐

Based on your previous experience, how much you would like to <u>DECREASE</u> each of the following emotions to get the most out of your musical practice?
(1 = Not at all; 7 = A great deal)

Happiness	☐
Energy	☐
Calmness	☐
Gloom	☐
Guilt	☐
Sluggishness	☐
Anxiety	☐
Downheartedness	☐
Nervousness	☐
Anger	☐

References:

Breaden Madden, G., and Jabusch, H-C. (2021). Instrumental and Hedonic Motives for Emotion Regulation in Musical Practice. *Front. Psychol.* 12:643974. doi: 10.3389/fpsyg.2021.643974
